# The Complete Chloroplast Genome of *Guadua angustifolia* and Comparative Analyses of Neotropical-Paleotropical Bamboos

**DOI:** 10.1371/journal.pone.0143792

**Published:** 2015-12-02

**Authors:** Miaoli Wu, Siren Lan, Bangping Cai, Shipin Chen, Hui Chen, Shiliang Zhou

**Affiliations:** 1 Forestry College, Fujian Agriculture and Forestry University, Fuzhou, 350002, Fujian, China; 2 Xiamen Botanical Garden, Xiamen, 361000, Fujian, China; 3 State Key Laboratory of Systematic and Evolutionary Botany, Institute of Botany, the Chinese Academy of Sciences, Beijing, 100093, China; Academia Sinica, TAIWAN

## Abstract

To elucidate chloroplast genome evolution within neotropical-paleotropical bamboos, we fully characterized the chloroplast genome of the woody bamboo *Guadua angustifolia*. This genome is 135,331 bp long and comprises of an 82,839-bp large single-copy (LSC) region, a 12,898-bp small single-copy (SSC) region, and a pair of 19,797-bp inverted repeats (IRs). Comparative analyses revealed marked conservation of gene content and sequence evolutionary rates between neotropical and paleotropical woody bamboos. The neotropical herbaceous bamboo *Cryptochloa strictiflora* differs from woody bamboos in IR/SSC boundaries in that it exhibits slightly contracted IRs and a faster substitution rate. The *G*. *angustifolia* chloroplast genome is similar in size to that of neotropical herbaceous bamboos but is ~3 kb smaller than that of paleotropical woody bamboos. Dissimilarities in genome size are correlated with differences in the lengths of intergenic spacers, which are caused by large-fragment insertion and deletion. Phylogenomic analyses of 62 taxa yielded a tree topology identical to that found in preceding studies. Divergence time estimation suggested that most bamboo genera diverged after the Miocene and that speciation events of extant species occurred during or after the Pliocene.

## Introduction

The chloroplast or plastid is a cellular organelle that is derived from a free-living cyanobacterial-like prokaryote; this event occurred by endosymbiosis ~1.5 billion years ago [1]. By interacting with the host genome and adapting to the cytoplasmic environment, plastids have undergone extensive genomic changes and have become indispensable to plant cells. One of the most remarkable changes to the original genome is the reduction of genome size; only ~5% of the original chloroplast genome remains [1, 2]. The chloroplast genomes of land plants range from 19 [3] to 218 kb [4] and, in most cases, exhibit a highly conserved quadripartite organization divided by a pair of inverted repeats (IRs) that separate the genome into a large single-copy (LSC) region and a small single-copy (SSC) region.

Although these conserved chloroplast genome structures are present in most plants, the chloroplasts of several angiosperms are an exception, having independently undergone substantial alterations. Gene loss and pseudogenization commonly occur during chloroplast evolution in parasitic plants [5, 6]. Such deletions of chloroplast DNA (cpDNA) sequences occur to various degrees, ranging from one or a few bases to whole genes or to one copy of the IR region [7, 8]. Some plants groups are amenable to large-scale rearrangements, including 90% of fern species [9], conifers [10] and the legume family [11]. Extensions or contractions of IR regions and gene loss also commonly occur during chloroplast genome evolution in angiosperms [12]. In addition, several cases of intracellular fragment acquisition by chloroplast genomes have been demonstrated in milkweeds, carrot (*Daucus carota*) [13] and five members of Bambusoideae [14, 15].

Because these structural mutations are accompanied by speciation over time, such informative changes are well suited to indicate evolution. The very nature of the chloroplast genome, including its small size, simple and conserved structure, tremendous evolutionary-rate variations among gene loci and abundant copies per cell, have rendered the chloroplast genome more useful in molecular evolutionary analyses and phylogenetic reconstruction than mitochondrial and nuclear genomes. Because the entire chloroplast sequence harbors complete and stronger phylogenetic signals compared with the small number of loci, chloroplast genomes have been extensively utilized for comparative and phylogenetic studies in association with large, closely related and globally distributed plant groups [16, 17], albeit the advantages of having a complete chloroplast genome sequence might be offset by lower sampling [18]. Given these characteristics, chloroplast genomes represent good models for testing lineage-specific molecular evolution.

Selective pressures lead plants to diversify as they adapt to new terrestrial landscapes. Chloroplast genomes bear the scars of these pressures by exhibiting different patterns of diversity in different environments. The evolution of smaller chloroplast genomes, for instance, tends to be associated with harsh environments [19]. Currently, little is known about the genetic processes that govern the evolution of closely related species that grow in the neotropics or paleotropics. Bamboos are among the most typical of plant species that occupy both the paleotropics and the neotropics and are members of the subfamily Bambusoideae of Poaceae. This subfamily has diverged into three distinct lineages that correspond to three tribes, i.e., Arundinarieae (temperate woody bamboos), Bambuseae (tropical woody bamboos) and Olyreae (herbaceous bamboos) [20]. Species in the Bambuseae tribe are usually subdivided into paleotropical and neotropical groups. By June 2015, 46 entirely sequenced chloroplast genomes from bamboos (25 from Arundinarieae, 10 from Olyreae, and 16 from Bambuseae) were available in the NCBI database [14, 15, 21–24]. Studies have analyzed full chloroplast sequences to understand the relationship between bamboo species. Here, we present an analysis of chloroplast genome evolution in representative paleotropical and neotropical bamboos using one new molecular data and other published chloroplast genomes. We report the sequence of the complete chloroplast genome of *Guadua angustifolia* Kunth, an economically and ecologically important neotropical bamboo that is found in Colombia, Ecuador and Venezuela. The main purpose of this study is to apply comparative genomics approaches to test the hypothesis that the chloroplast genomes of paleotropical and neotropical species have evolved differently due to differences in the selective forces between continents.

## Materials and Methods

### DNA extraction, genome sequencing and assembly

Leaves of *G*. *angustifolia* were harvested in the Hua’an Bamboo Garden, Fujian Province, China (25° 00′ 52.07″ N, 117° 32′ 04.13″ E; the plant was introduced from Ecuador in 2001). *G*. *angustifolia* is not an endangered or protected species, and the sample collection was approved by the Forestry Bureau of Hua’an County.

Total DNA was isolated from silica gel-dried leaves using the mCTAB method [25]. The chloroplast genome was amplified using 138 universal primer pairs, which were provided by Dong et al. [26]. The fragments were then sequenced using the Sanger method. All sequences were proofread and assembled using Sequencher 4.7 software (Gene Codes Corporation, Ann Arbor, Michigan, USA). Gaps were bridged by sequences that were amplified using *G*. *angustifolia*-specific primers (S1 Table). The complete chloroplast genome was finally assembled from 226,314 bp of sequence data, representing approximately 1.7-fold coverage of the *G*. *angustifolia* chloroplast genome. The genomic structure was further verified following the method of Dong et al. [26].

### Genome annotation and visualization

The resulting chloroplast genome was annotated using DOGMA [27]. Using this web-based tool, start/stop codons and intron/exon boundaries were adjusted after BLASTX searching (e-value cutoff = 1e-10) for homologous genes against a custom database of 16 previously published chloroplast genomes. The obtained tRNA genes were further confirmed using DOGMA and tRNAscan-SE [28]. The GenomeVx program [29] was applied to graphically display the physical genetic loci within the chloroplast genome.

### Structural and sequence variations among the chloroplast genomes of alliances

The chloroplast genomes of the herbaceous bamboo *Cryptochloa strictiflora* (E.Fourn.) Swallen and the paleotropical woody bamboo *Dendrocalamus latiflorus* Munro were compared to that of *G*. *angustifolia* (the reference species). Variations in gene contents and orders were then visualized using MultiPipmaker (http://pipmaker.bx.psu.edu/pipmaker/). The relative evolutionary rates of the protein-coding sequences of the three chloroplast genomes were quantified based on nonsynonymous (dN) and synonymous (dS) substitutions and their ratios (ω = dN/dS); *Ferrocalamus rimosivaginus* was used as an outgroup. dN and dS were computed according to the Nei and Gohobori method as implemented in the PAML package v4.4 [30] using the F3×4 codon-based substitution model. Only codons shared among all chloroplast genomes were compared. Pseudogenes (*ndhH* and *ycf68*) were not included in the analyses. dN, dS and ω were calculated for (1) individual protein-coding genes; (2) all protein-encoding genes combined; and (3) groups of genes with the same functions, e.g., photosynthesis ATP-synthase genes or NADH-dehydrogenase genes. Tajima’s relative rate test, as implemented in MEGA 5 [31], was applied to determine the constancy of evolutionary rates among the three bamboos. Differences between estimated dN and dS values among gene groups within each species were tested for significance using the Kruskal-Wallis test (SPSS v.18.0; SPSS Inc., Chicago, IL,USA).

### Sequence alignment and phylogenomic inference

To determine the phylogenetic position and divergence time of *G*. *angustifolia*, 61 complete chloroplast genomes, representing 38 genera of Poaceae (S2 Table), were obtained from GenBank and aligned with the chloroplast genome of *G*. *angustifolia*. Based on APG III [32], *Anomochloa marantoidea* Brogn. was defined as the outgroup. Multiple alignments were generated for full-length chloroplast genomes using Clustal X [33] followed by manual correction with Se-Al [34]. IRa, long gaps introduced in one or several sequences and unreliably aligned regions were excluded from the analyses. The remaining gaps were treated as missing data. Phylogenomic trees were constructed using maximum parsimony (MP), maximum likelihood (ML) and Bayesian methods. MP analysis was conducted using PAUP version 4.0 b10 [35]. Parsimony heuristic searches were performed with TBR branch swapping using 1,000 random sequence-addition replicates, and bootstrap support was obtained from 1,000 replicates. ML phylogenetic analysis was performed using RAxML [36] and 1,000 bootstrap replicates. The GTR+G+I model was chosen using Modeltest v 3.7 [37]. Bayesian inference (BI) was conducted using MrBayes 3.2.2 [38]. One tree was sampled every 1,000 generations for 5,000,000 generations until the average standard deviation of split frequencies fell below 0.01, with 25% as burn-in. The ML and BI analyses were run on the CIPRES Science Gateway V.3.3 [39].

### Divergence time estimation

A Bayesian dating analysis was performed using the BEAST v 1.7.3 package [40] to estimate the divergence time of the focal bamboo species and of other Poaceae lineages. To lower the computing burden, we constructed a reduced dataset of 46 chloroplast genomes by retaining only one representative chloroplast genome per genus. The input file for the analyses was prepared using BEAUti. BEAST runs were conducted using the model GTR+G+I with an uncorrelated lognormal relaxed clock model, a Yule prior on speciation, and relative priors on divergence times. The molecular tree was calibrated by (1) placing a 90–115 Ma [41, 42] constraint on the crown group of Poaceae, (2) setting the split of *Oryza* and *Leersia* between 5–34.5 Ma [43], and (3) placing a minimum of 3.12 Ma and a maximum of 5.3 Ma on *Guadua* based on the existence of macrofossils in the Pliocene [44, 45]. Posterior distributions of parameters were approximated using MCMC analyses of 7 separate runs of 50 million generations. One tree was sampled every 1,000 generations, and the first 25% of the sampled trees was discarded as burn-in. All outputs were inspected in Tracer v 1.5 (http://www.beast.bio.ed.ac.uk/) to verify that the effective sample size (ESS) statistic exceeded 200 for all estimated parameters. The maximum clade credibility tree was summarized using TreeAnnotator v1.7.3. BEAST analyses were also performed at the CIPRES Science Gateway V.3.3 [39].

## Results

### Genome feature for *G*. *angustifolia*

**Fig 1:**
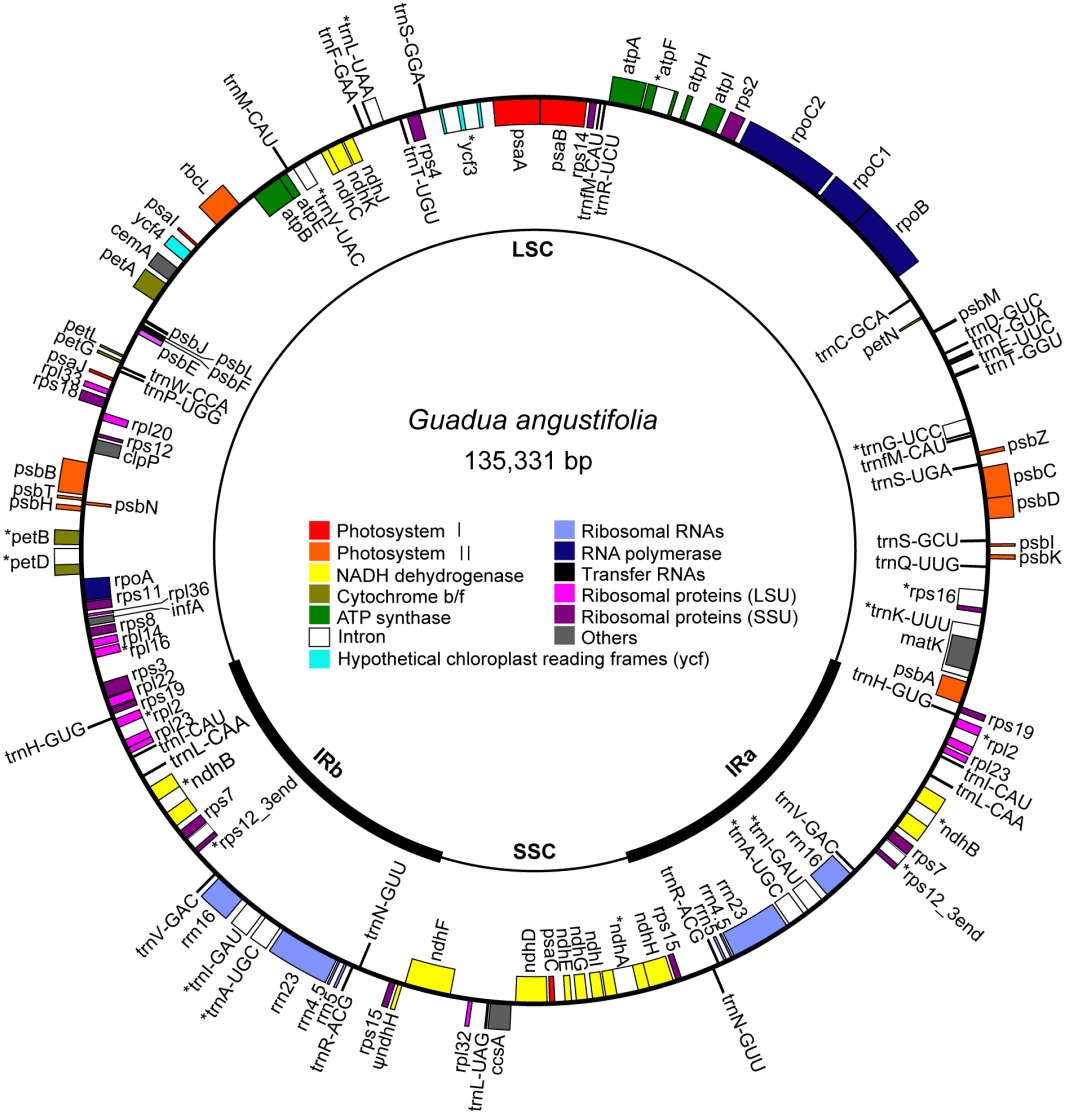
Gene map of the *Guadua angustifolia* chloroplast genome. Inner thick arcs represent the inverted repeat regions (IRa and IRb). Genes inside and outside of the circle are transcribed clockwise and counterclockwise, respectively. Genes are colored according to their functional groups. *: genes with intron(s). Ψ: pseudogenes.

The complete *G*. *angustifolia* chloroplast genome (KM365071) exhibited the conventional chloroplast quadripartite structure of land plants and mapped as a single circular double-stranded DNA molecule of 135,331 bp (Fig 1). The chloroplast genome harbored an LSC region of 82,839 bp, an SSC region of 12,898 bp, and two IR regions of 19,797 bp each. The AT content of the entire genome was 61.3%. The AT contents of the various genomic components were as follows: 60.6% in protein-coding genes, 48.0% in tRNA genes, 45.3% in rRNA genes, 61.6% in introns, and 65.2% in intergenic spacers.

There were 112 unique genes in the *G*. *angustifolia* chloroplast genome, including 77 protein-coding genes, 31 tRNA genes and 4 rRNA genes. Nineteen genes were duplicated in the IR regions, yielding a total of 131 genes in this newly determined chloroplast genome. A total of 60 protein-coding genes and 22 tRNA genes were distributed within the LSC, and 10 protein-coding genes and 1 tRNA gene were present within the SSC. Among the 15 intron-containing genes, only the *ycf3* gene contained two intervening introns; the remaining genes contained one intron each. The *rps12* gene comprised three exons; one exon fell into the LSC region, and the remaining two exons were duplicated in the IR regions. The IR/SSC boundary lay in the *ndhH* gene, resulting in part of *ndhH* being duplicated in IRb as a pseudogene.

The architecture, gene content and AT content of the *G*. *angustifolia* chloroplast genome resembled those of other published tropical and temperate bamboos (Table 1). In particular, the two congeneric species of *Guadua* showed greater chloroplast genomic similarities, with a total of 153 sequence variations (110 SNPs, 41 indels and two inversions). Amongst the 130 SNPs detected, 57% corresponded to transitions and 43% to transversions. Of the 41 indels, 27 were mononucleotide repeats, and the remainders were small indels (5–27 bp) that were present in noncoding regions (*rps16*-*trnQ*, *trnS*-*psbZ*, *psbZ*-*trnG*, *psbM*-*petN*, *petN*-*trnC*, *trnC*-*rpoB*, *atpF* intron, *ycf3* intron, *rbcL*-*psaI*, *petD*-*rpoA*, *rps15*-*ndhF*, *ndhF*-*rpl32*).

**Table 1:** Global features of bamboo chloroplast genomes.

Features	Size (bp)	LSC (bp)	SSC (bp)	IRs (bp)	Spacer length (bp)	Intron length (bp)	AT content (%)	No. of genes	No. of introns
CS	135,033	80,556	13,465	20,506	46,893	15,985	61.1	127	16
DL	139,394	83,011	12,875	21,754	51,656	15,988	61.1	128	16
GA	135,331	82,839	12,898	19,797	46,846	15,960	61.3	128	16
GW	135,324	82,806	12,932	19,793	47,095	15,985	61.2	128	16
AP	139,697	83,273	12,834	21,795	51,245	15,968	61.1	128	16
AG	138,935	82,632	12,711	21,796	50,507	15,924	61.1	128	16
BE	139,493	82,988	12,901	21,802	50,925	15,975	61.1	128	16
FR	139,467	83,091	12,718	21,829	51,256	16,017	61.2	128	16
IL	139,668	83,273	12,811	21,792	51,143	15,926	61.1	128	16
PE	139,679	83,213	12,811	21,798	51,195	15,991	61.1	128	16
TL	161,572	89,140	19,652	26,390	52,898	18,118	66.2	131	18

CS: *Crytochloa strictiflora*;

DL: *Dendrocalamus latiflorus*;

GA: *Guadua angustifolia*;

GW: *Guadua weberbaueri*;

AP: *Acidosasa purpurea*;

AG: *Arundinaria gigantea*;

BE: *Bambusa emeiensis*;

FR: *Ferrocalamus rimoslvaginus*;

IL: *Indocalamus longiauritus*;

PE: *Phyllostachys edulis*;

TL: *Typha latifolia*.

These genomic characteristics were quite common even when compared with other grasses [46]. Further comparison with the early diverging Poales *T*. *latifolia* indicated that all Poaceae chloroplast genomes had relatively compact sizes (~135–139 kb vs ~161 kb). Additionally, as reported in previous studies, [47], features unique to Poaceae were observed, such as the loss of the *ycf1*, *ycf2* and *accD* genes, as well as loss of the introns of *clpP* and *rpoC1*. In addition, three DNA inversions between *trnS* ^GCU^ and *trnfM* ^CAU^ within the LSC region were found in all Poaceae species.

### Chloroplast genome comparison among *G*. *angustifolia*, *Dendrocalamus latiflorus* and *Cryptochloa strictiflora*

**Fig 2:**
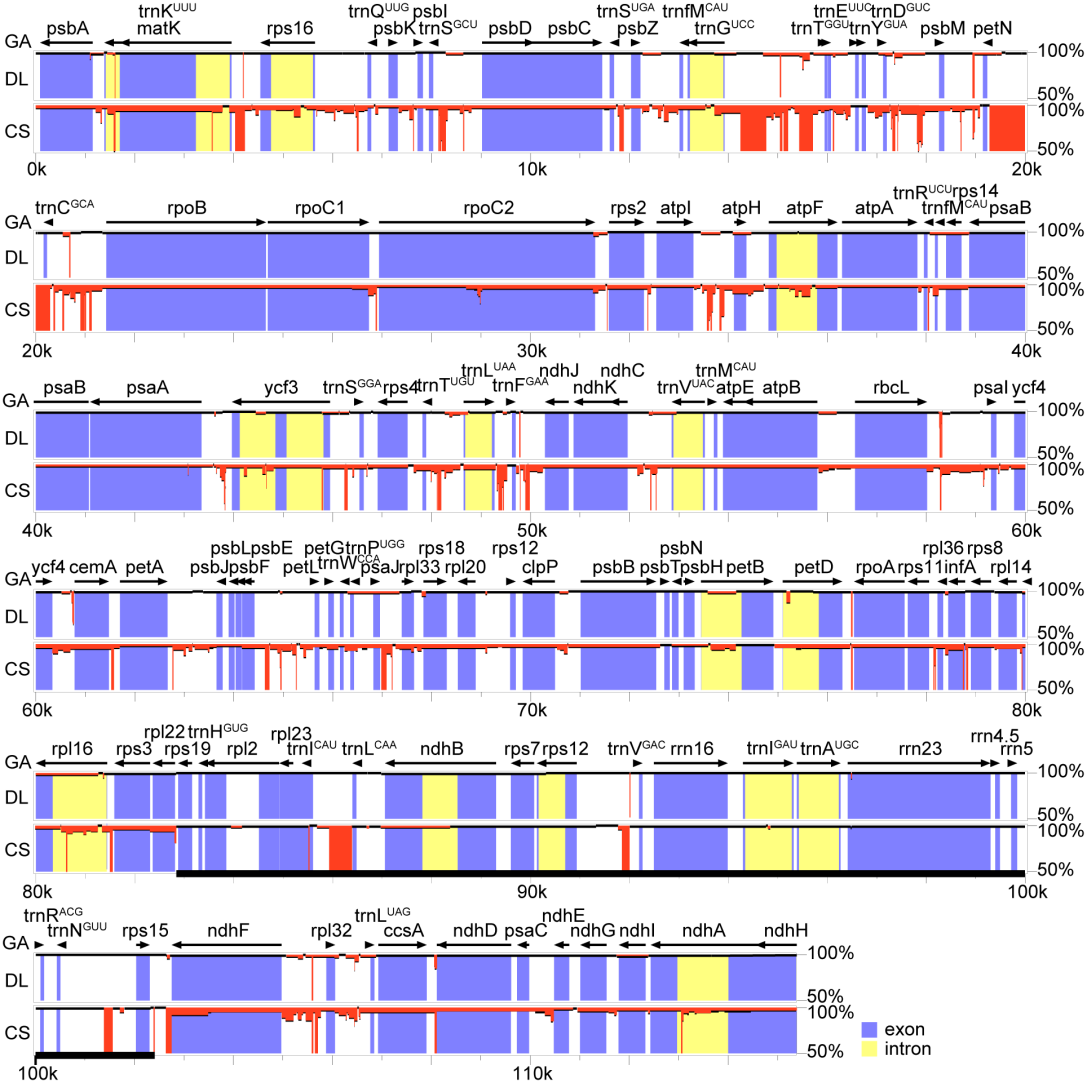
Whole plastid genome alignments for the three studied bamboos. *Guadua angustifolia* was used as a reference and is presented as the uppermost line; gene orientations are shown as arrows. IRa was removed from the alignment, and IRb is underlined in bold. Sequence similarities of less than 100% are highlighted in red. GA: *Guadua angustifolia*; DL: *Dendrocalamus latiflorus*; CS: *Cryptochloa strictiflora*.

Sequence differences among the three representative tropical bamboos (*G*. *angustifolia*, *D*. *latiflorus* and *C*. *strictiflora*) were visually quantified using “percent identity plots”the annotation of *G*. *angustifolia* was used as a reference (Fig 2). Global alignments revealed perfect synteny among the three chloroplast genomes, and *C*. *strictiflora* presented a higher level of sequence divergence. Overall, woody *G*. *angustifolia* shared 98.7% sequence similarity with *D*. *latiflora* and 95.1% similarity with *C*. *strictiflora*. The IRs were more conserved than the LSC and SSC. Variations appeared more abundant in noncoding regions (intergenic spacer regions and introns) than in coding regions. Mutational hotspot regions in the alignment were also identified and encompassed the *trnG-trnT*, *trnD-psbM*, *rpl32*-*ccsA*, and *ndhF-rpl32* regions. Other evolutionary differences among the three chloroplast genomes were mainly inferred from genome size, IR expansion and contraction, and the pseudogene at the IRb/SSC border.

#### Genome size

The *G*. *angustifolia* chloroplast genome was 4,063 bp smaller than that of the paleotropical bamboo *D*. *latiflorus* and was slightly larger than that of herbaceous bamboo *C*. *strictiflora* (Table 1). The lengths of the LSC and SSC differed little between the two woody bamboos despite the distinct difference in the overall size of their chloroplast genomes. However, a much shorter LSC and a longer SSC, 80 kb and 13 kb, respectively, were observed for the herbaceous bamboo *C*. *strictiflora*.

The chloroplast protein synthesis apparatus was almost identical in the three bamboos, except that *C*. *strictiflora* possessed only one copy of the *rps15* gene due to a shifted IR/SSC border (138 bp). Nevertheless, coding regions and introns remained largely invariant; the total coding region varied from 75,458 bp (*D*. *latiflorus*) to 75,888 bp (*G*. *angustifolia*), whereas the total intron length varied from 14,064 bp (*G*. *angustifolia*) to 14,135 bp (*C*. *strictiflora*).

The genome size differences between the neotropical and paleotropical bamboos were primarily due to length variations in the intergenic spacers that resulted from insertion/deletion changes. In comparison with *D*. *latiflorus*, an exceptional IR-located 1,521-bp deletion in the *trnI-trnL* spacers and a 461-bp deletion in *rps12-trnV* were found in *G*. *angustifolia*. These unusual intergenic deletions clearly led to the decreased genome size of *G*. *angustifolia*. When the intergenic spacers of *G*. *angustifolia* were contrasted with those of *C*. *strictiflora*, 12 intergenic spacers were found to have notable length differences >100 bp, 7 of which were expanded and 5 were diminished. Taken together, these length differences accounted for the slight variations in the total length of the spacing regions of these two chloroplast genomes (46,938 bp for *G*. *angustifolia* and 46,846 bp for *C*. *strictiflora*).

#### IR expansion and contraction and gene pseudogenization

**Fig 3:**
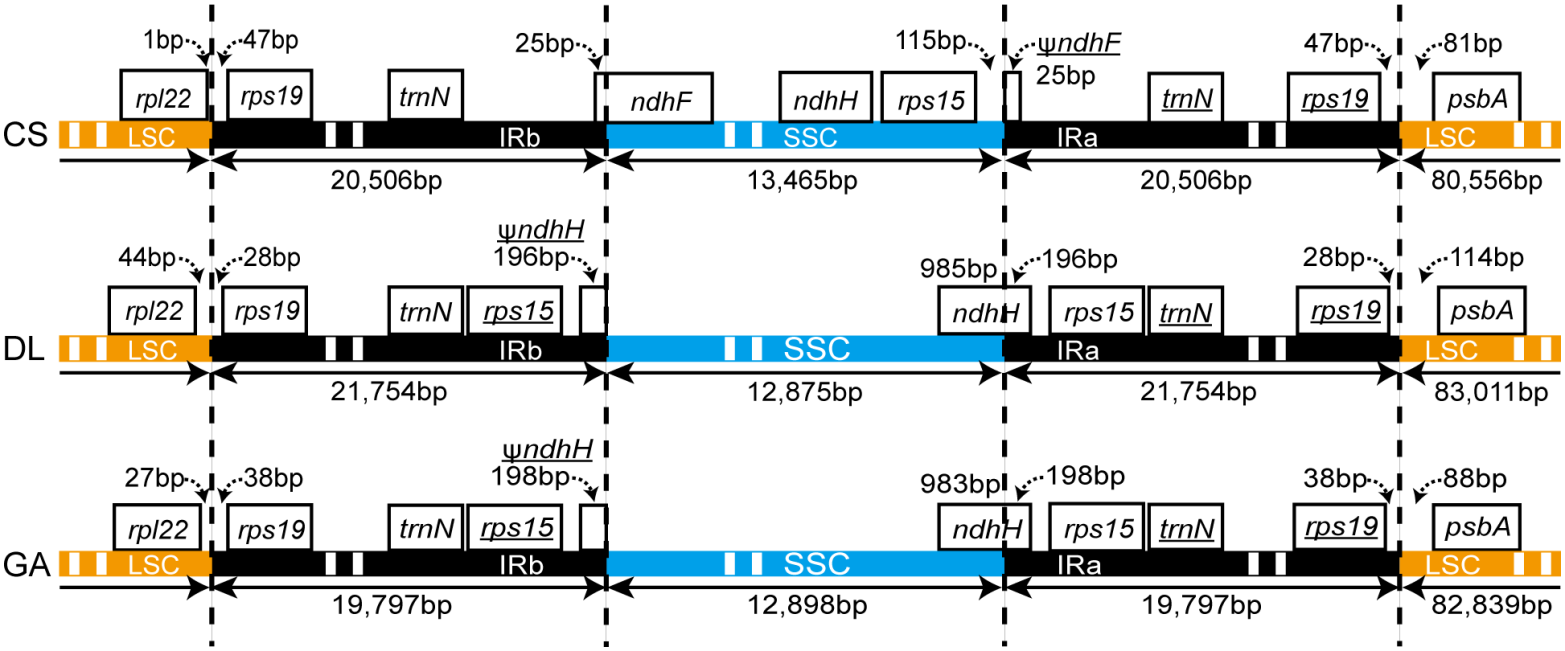
Comparison of IR boundaries among the three studied bamboo chloroplast genomes. Underlined genes are transcribed counterclockwise. CS: *Cryptochloa strictiflora*; DL: *Dendrocalamus latiflorus*; GA: *Guadua angustifolia*.

Expansion and contraction of IR regions led to differences at the IR borders among the three complete bamboo chloroplast genomes, as shown in Fig 3. Complete *rps19* genes resided in the IRs at varying distances to the IRb/LSC junction (47 bp, 28 bp and 38 bp in *C*. *strictiflora*, *D*. *latiflorus* and *G*. *angustifolia*, respectively). Although the IR regions of *G*. *angustifolia* (19,797 bp) were much shorter than those of *D*. *latiflorus* (21,754 bp), *G*. *angustifolia* and *D*. *latiflorus* were quite similar at the IR/SSC boundary. The *ndhH* gene, which is encoded by 196 bp in *D*. *latiflorus* and by 198 bp in *G*. *angustifolia*, straddled the IRa/SSC junction and generated an incomplete copy (Ψ*ndhH*) in the IRb of both chloroplast genomes.

The IR/SSC junction locations were slightly disparate in herbaceous bamboo *C*. *strictiflora*. The IR region of *C*. *strictiflora* had contracted; thus, the junction of IRb/SSC falls into the *ndhF* gene. This contraction also causes the *rps15* and *ndhH* genes to occur as single-copy genes within the SSC rather than in the IR regions, as observed in the two woody bamboos. Consequently, this phenomenon generated the other pseudogene Ψ*ndhF* at the IRa/ SSC boundary region and reduced the total number of *C*. *strictiflora* genes to 127.

The *ycf68* gene that was nested in the *trnI* ^GAU^ intron in the IR regions appeared to be a complete 405-bp open reading frame in the woody bamboo *G*. *angustifolia* and *D*. *latiflorus* but was much shorter in the herbaceous bamboo *C*. *strictiflora*. The 45th codon was identified as a stop codon that disrupted the CDS; thus, *ycf68* is likely to be a pseudogene in *C*. *strictiflora*.

### Evolutionary rates of neotropical-paleotropical chloroplast genomes

Using the *F*. *rimosivaginus* chloroplast genome as a reference, the dN, dS and ω of 76 protein-coding genes in *G*. *angustifolia*, *D*. *latiflorus* and *C*. *strictiflora* were computed and compared (Table 2, S3 Table). Unsurprisingly, rate heterogeneity existed among lineages, gene groups and genes. For all the coding genes combined, the mean dN and dS values were generally higher in *C*. *strictiflora* (0.0148±0.0006 and 0.0823±0.0027, separately) than in *G*. *angustifolia* or *D*. *latiflorus*. *G*. *angustifolia* and *D*. *latiflorus* showed similar dN and dS rates (Kruskal-Wallis tests; P = 0.278 for dN, P = 0.631for dS); the value of ω was slightly higher in *D*. *latiflorus*, revealing similar evolutionary rates between the paleotropical and neotropical woody bamboos. Relative rate tests further confirmed that the substitution rates were statistically higher in most of the *C*. *strictiflora* genes analyzed (49 out of 76 genes) (P<0.001). Only in *C*. *strictiflora* was there a slight correlation between dN and dS across genes (Pearson’s r = 0.25, P = 0.0315).

**Table 2:** Average substitution rates of 76 concatenated protein-coding genes in the three studied chloroplast genomes.

Taxa	Nonsynonymous (dN)	Synonymous (dS)	dN/dS (ω)
***C*. *strictiflora***	0.0148±0.0006	0.0823±0.0027	0.1801
***D*. *latiflorus***	0.0057±0.0004	0.0271±0.0014	0.2091
***G*. *angustifolia***	0.0053±0.004	0.0297±0.0015	0.1776

*F*. *rimosivaginus* is used as a reference. Data are showed as the means ± standard errors.

**Fig 4:**
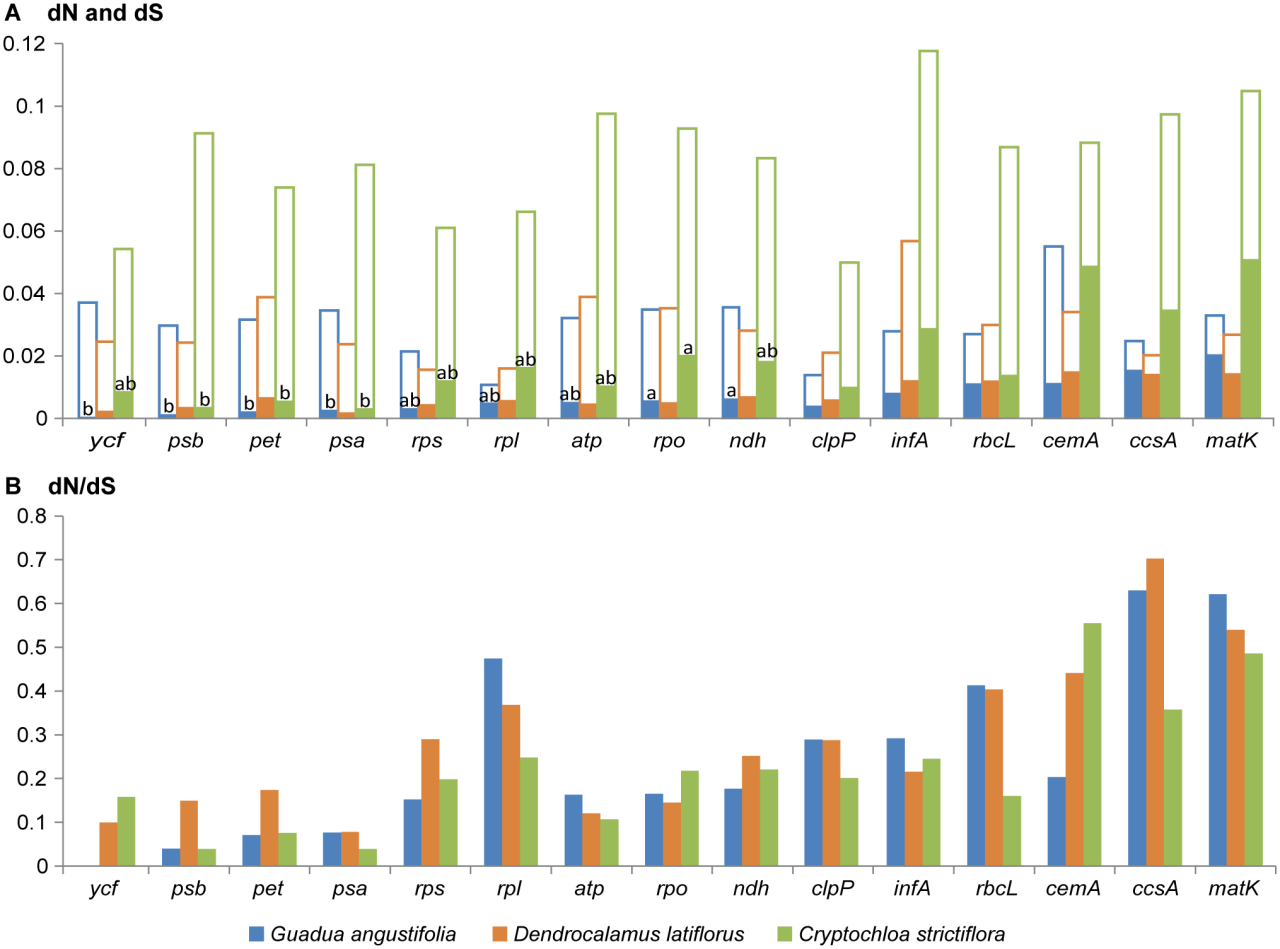
Nonsynonymous substitution (dN), synonymous substitution (dS), and dN/dS values for individual genes or gene groups. (A) dN and dS values are shown as overlapping bars; hollow bars indicate dS values, and solid bars indicate dN values. (B) dN/dS values.

After sorting the genes into functional categories, significant differences were revealed among the groups. The *cemA matK* and *ccsA* genes exhibited the highest dN and ω values, whereas dS was highest in *infA* (0.0567) (Fig 4A). Higher ratios of dN and dS indicated that the *cemA*, *matK*, *ccsA*, *rbcL* and ribosomal protein genes were selected for sequence diversity, and the *psb* and *psa* genes were conserved to a greater extent than the other gene groups (Fig 4B). Among the gene categories, the mean dN varied in *C*. *strictiflora* and *G*. *angustifolia* (Kruskal-Wallis test, P<0.05), whereas the mean dS values were uniform in each bamboo examined. Not all gene groups contained sufficient numbers of genes to apply multiple comparison tests, which require at least two data points in each group. Thus, the Student Newman Keuls (S-N-K) multiple comparison tests were performed on the first 9 groups shown in Fig 4. In both cases exhibiting significantly different dN values, *psb*, *pet* and *psa* presented the smallest mean dN values.

A comparison among individual genes in each functional group (S4 Table) showed that substitution rates fluctuated widely among the 76 genes, with dN values ranging from 0 to 0.05 and dS values ranging from 0 to 0.263. The highest dN value was detected in *matK*, and the highest dS value was detected in *rpl36*. Most genes exhibited dN/dS ratios of less than 0.5, indicating the efficiency of purifying selection. Two genes in *D*. *latiflorus*, *rpl32 and rpl33*, both of which were functionally essential in translation activity, exhibited dN/dS ratios that were significantly greater than unity (1.2009 and 1.3791).

### Phylogenomic position of *G*. *angustifolia* and age estimates

**Fig 5:**
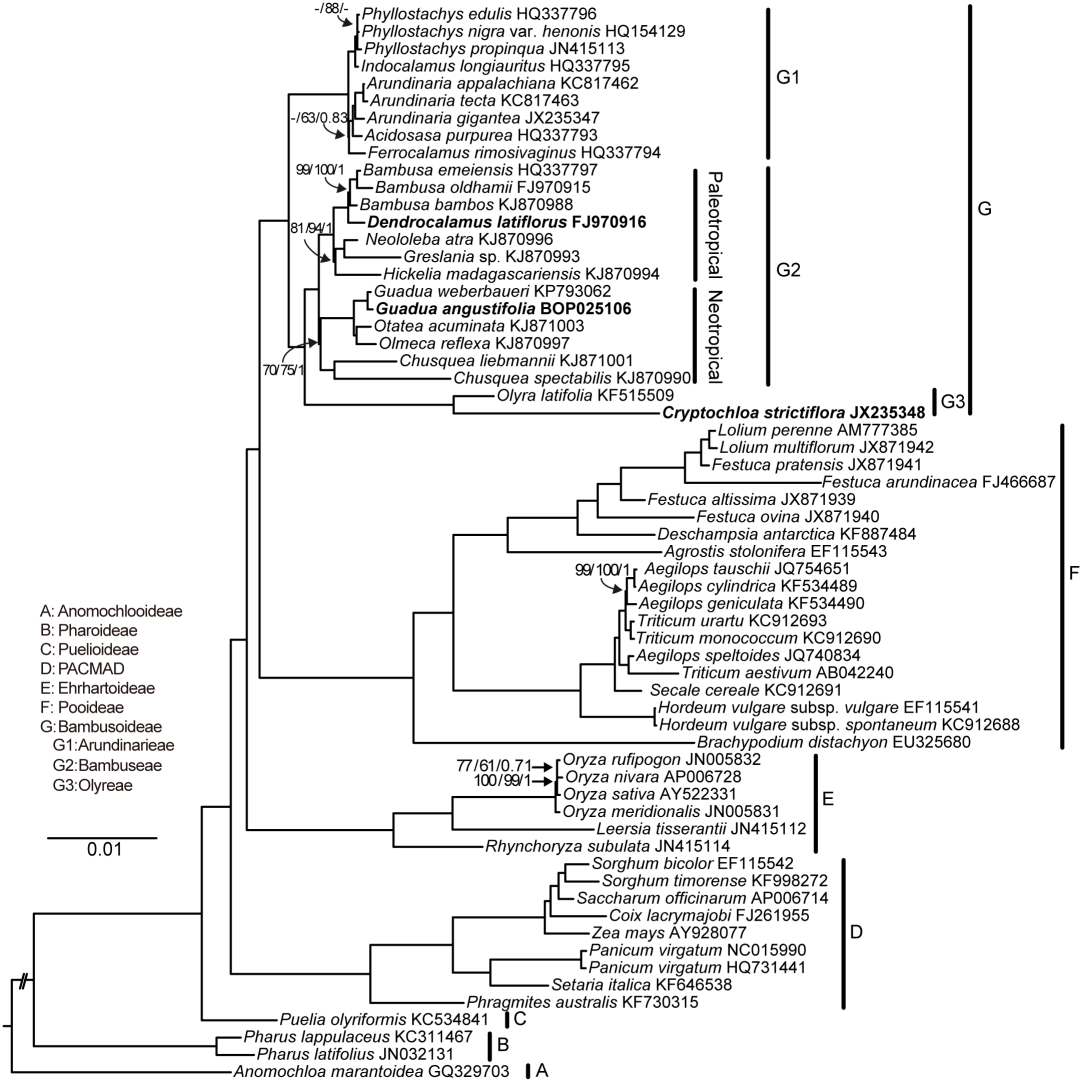
Maximum likelihood (ML) tree for 62 taxa based on completely aligned chloroplast genomes. IRb and large gaps were excluded from the analysis. All nodes have 100% bootstrap percentages (BS) and 1.0 Bayesian posterior probabilities (PP), except where noted. Support values are shown as MP bootstrap/ ML bootstrap/ Bayesian posterior probability. “-” indicates nodes that are not supported in the analysis. The three tropical chloroplast genomes examined in this study are indicated in bold.

A dataset of 113,641 unambiguously aligned nucleotide characters was finally subjected to phylogenomic analysis. MP, ML and BI analyses recovered almost identical and strongly supported relationships among the major clades (Fig 5). The phylogenomic tree identified seven clades corresponding to six subfamilies and a PACMAD group. The subfamily Bambusoideae (Clade G), which was united with Pooideae, formed a sister group to Oryzoideae. Within Bambusoideae, all three trees showed similar topologies with strong bootstrap support. The subfamily has diverged into two major subclades. Subclade G1 comprises temperate woody bamboos classified as Arundinarieae, and Subclade G2 comprises tropical woody and herbaceous bamboos that are classified into Bambuseae and Olyreae, respectively. The tropical herbaceous bamboos are sisters to the woody bamboos, and the neotropical bamboos are sisters to the paleotropical bamboos. A long branch leading to the herbaceous bamboos indicated accelerated rates of evolution. As expected, the newly sequenced *G*. *angustifolia* was grouped with its congener *G*. *weberbaueri*, and both species share a phylogenetic neighborhood with other neotropical bamboos.

**Fig 6:**
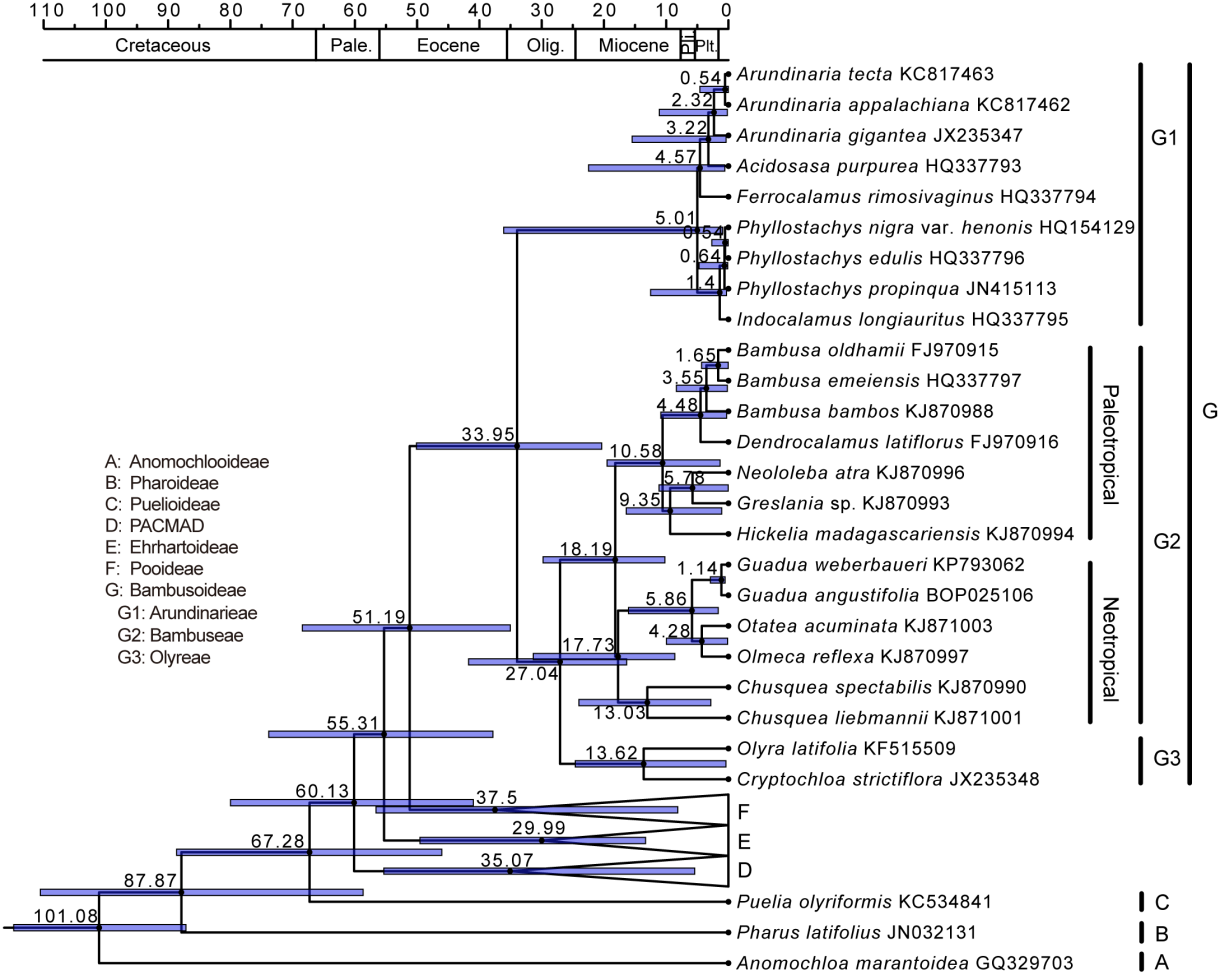
Bayesian chronogram for Bambusoideae and related subfamilies. Estimates of divergence times for major clades are listed near nodes and blue bars show the 95% highest posterior density (HPD) of relevant nodes. Pale., Paleocene, Olig., Oligocene, Pli., Pliocene, Plt., Pleistocene.

Initial divergence dates of the major clades within Poaceae were estimated based on entire chloroplast genome data (Fig 6). The divergence times of Poaceae major lineages primarily fell within the Paleocene and the Eocene (S5 Table). Bambusoideae diverged from Pooideae during the Eocene. The split between temperate and tropical bamboos likely occurred during the Oligocene. The temperate bamboos radiated from the late Pliocene. The herbaceous bamboos diverged from their woody counterparts during the late Oligocene (16.34–41.75 Ma 95% highest posterior density, HPD), and the neotropical woody bamboos diverged from the paleotropical woody bamboos during the Miocene (HPD: 10.21–29.77 Ma).

## Discussion

### The conserved chloroplast genome of bamboos

Complete sequencing and comparative analyses demonstrated that the neotropical *G*. *angustifolia* chloroplast genome bears a high level of conservation in terms of architecture and linear sequence order with other members of Bambusoideae. The most conspicuous difference appeared in chloroplast genome size when *G*. *angustifolia* was compared to its woody relatives. With the smallest IR size known in bamboos (19,797 bp), a ~3 kb smaller chloroplast genome was present in *G*. *angustifolia* (Table 1). The relative size of IRs varies significantly among angiosperm. Such changes are generally shaped by frequent contraction/expansion into or out of single-copy regions [12].In contrast, large intergenic sequence deletions in the IR region were ultimately responsible for the small IR size of *G*. *angustifolia*. Despite their differences in size, the gene content of the IRs remained identical. Exceptions included the *rps15* gene and genes located in the SSC-end of the IR; accompanying the IR/SSC boundary shift, woody bamboos organized *rps15* and a portion of the *ndhH* gene in each of the IRs, whereas the *rps15* gene of *C*. *strictiflora* was fully confined to the SSC, and a part of *ndhF* resided in the IRs (Fig 3). Previously, both forms of IRs were reported in the grass family as symplesiomorphic states [48]. The *C*. *strictiflora*-like IR, which also emerged in taxa of the PACMAD clade, supports the idea that IR/SSC borders evolved independently in the grass family and was not consistent with the observed phylogenetic relationships.

The functions of hypothetical gene *ycf68* are ambiguous in different land plant types, probably because the >400-bp open reading frame of *ycf68* can be read through without any internal stop codon in the chloroplast genomes of some plants but not in others. In woody bamboos, the *ycf68* locus appeared to be intact. However, in the herbaceous bamboo *C*. *strictiflora*, the identification of the frameshifting change and stop codon approximately one-third of the way into the *ycf68* region suggests that this sequence is a pseudogene. Thus, this result added new evidence confirming the suggestion by Raubeson et al. [49] that *ycf68*, although conserved, appears to be nonfunctional because of its location in the generally conserved IR region.

### Whole chloroplast genome phylogeny of bamboos

Although some bamboos occur in subtropical or even temperate regions, the great majority of bamboos occupy tropical and semi-tropical areas [50]. Bamboos have traditionally been classified into two tribes, Bambuseae (woody) and Olyreae (herbaceous), on the basis of morphological characteristics. Woody bamboos were further subdivided into temperate woody and tropical woody bamboos [51]. However, subsequent molecular phylogenetic studies based on multiple cpDNA fragments [20, 52] revealed a different scenario; in the new understanding, woody bamboos do not represent a monophyletic group because temperate bamboos form a sister clade to tropical bamboos. This classification scheme is supported by our phylogenomic analyses (Fig 5) and by the results of Burke et al. [21]. Recent analyses of three single-copy nuclear genes [53] offered additional evidence that woody bamboos originated through hybridization between five lineages in this group and that the paraphyly supported by chloroplast markers was likely an artifact. Conflict between chloroplast and nuclear phylogenetic trees suggests different patterns of evolution among sequence sets. Chloroplast genes are uniparentally inherited as a linked unit; therefore, chloroplast capture or lineage sorting might have occurred following the early hybridization of woody bamboos. In contrast, nuclear genes evolve independently through recombination. Therefore, single-copy nuclear loci that reflect similar evolutionary processes might provide true phylogenetic signals. In Triplett’s study [53], it was evident that one of the markers still implied the potential grouping of herbaceous and tropical woody bamboos. Because bamboos form a perplexing group that is noted for hybridization and polyploidy, with recent diversification, the chloroplast inference reported here only represents the maternally inherited signals. More robust inferences regarding bamboo history require the use of additional independent nuclear markers or other lines of evidence, such as mitochondrial phylogenetic markers and data sets obtained from ESTs.

### Evolutionary rates of neotropical-paleotropical bamboos

Because the nonsynonymous and synonymous substitution rates might indicate the constraints of natural selection on organisms, estimation of these mutations plays a pivotal role in understanding the dynamics of molecular evolution [54]. Substitution rate estimations indicate that genes of neotropical-paleotropical bamboos have evolved at variable rates, and patterns of substitution differ both in functional categories and lineages. Based on our knowledge of gene groups, variable rates have been proposed to link with a number of factors, such as relaxed or positive selection, functional constraints, and gene expression level [55]. However, distinguishing the prevailing factor governing locus-specific molecular evolution might prove difficult. As suggested by studies on the photosynthetic plants Geraniaceae [56], which showed accelerated substitution rates in ribosomal protein genes, rate variations might be due to differences in faulty DNA repair and in gene expression patterns. The former might be less likely given the perfect sequence synteny (no rearrangement) among the three bamboos (Fig 2). Alternatively, the hypothesis that gene expression level and substitution rate are negatively correlated is attractive because the same trend appears to be true in *Marchantia polymorphs* and *Nicotiana terbium* [57]. According to our data, the mutation rates of the *psa* and *psb* genes were less variable than those of the *ccsA*, *matK*, *cemA* and *rpl* genes, as evidenced by the lower dN/dS ratios obtained (Fig 4B, S4 Table). These rate patterns were consistent, to some extent, with Geraniaceae [56] and other grass lineages [58]. Although we do not have data on chloroplast gene expression in bamboos, gene expression levels could potentially affect the variable substitution rates among gene groups. Genome-scale analyses of gene expression would be required to advance our understanding of this point.

Early phylogenetic analyses suggested that herbaceous bamboos have evolved more rapidly than woody bamboos [59, 60]. By the same token, in our phylogenomic comparison, the herbaceous bamboo *C*. *strictiflora* was observed to display a longer phylogenetic branch length, which indicates an overall rate acceleration. Similarly, in the herbaceous *C*. *strictiflora*, the accumulation of mutations at both nonsynonymous and synonymous sites occurred at approximately twice the rate of its woody bamboo homologs (Table 2), a result supported by relative rate tests (P<0.05). Our analyses also show a strong correlation between dN and dS values in *C*. *strictiflora* (Pearson’s r = 0.25, P = 0.0315), hinting at a lineage-specific effect on this species. Because *C*. *strictiflora* is a herbaceous perennial that flowers annually [61], the higher overall substitution rates in the herbaceous bamboo *C*. *strictiflora* clearly support the general observation that molecular evolutionary rates are negatively scaled with generation time [62, 63]. It is worth noting that the rates of 27 loci that were estimated using Tajima's rate test were not significantly different from those obtained in woody bamboos (S3 Table). However, when looking more closely at the Nei and Gohobori results for each of the 27 genes, either the dN or dS of *C*. *strictiflora* (S4 Table) remained above those of woody species. This finding suggests that although relative rates failed to reject rate constancy for some genes among the three bamboos, the overall mutation rate of DNA evolution in herbaceous *C*. *strictiflora* is primarily generation-time dependent. Tajima’s test lacks the statistical power to detect moderate levels of rate variation for sequences of closely related species [64]. This is a plausible explanation for the somewhat discrepant results of the two methods.

Excluding *C*. *strictiflora*, few differences remained between the two woody bamboos in terms of sequence evolutionary rates. Both the dN and dS values of *G*. *angustifolia* were rather similar to those of *D*. *latiflorus* across all coding genes (Kruskal-Wallis tests; P = 0.278 for dN, P = 0.631 for dS), indicating that the selective constraints on the coding genes of these two species were comparable. These bamboos are both giant woody species with long generation times, and both exhibit little or no changes in structural organization of the chloroplast genomes; these observations might partly explain why these species evolved at similarly low rates. Based on our chloroplast-level divergence time estimates (Fig 6), the exact age of the neotropical bamboo *G*. *angustifolia* and the paleotropical bamboo *D*. *latiflorus* could not be strictly inferred due to incomplete sampling. The nodal 95% HPD of the most recent common ancestor for *G*. *angustifolia* and *D*. *latiflorus* greatly overlapped, possibly reflecting that they evolved over a similar period of time. Thus, identical and more recent divergence times might also explain the similar evolutionary rates between these two woody bamboos. Although strong selective pressures act against broad variations in functional genes, intergenic spacers might lack such constraints. We found a strong geographical link with the sequence evolution of intergenic spacers. Bamboos that grow in the New World consistently shared a very low probability of indel character in intergenic spacers. These indel mutations, although occurring in different regions among bamboos, were the principal factors shaping the chloroplast genome shrinkage. Also of note, such large length variations in intergenic spacers were commonly found within the BEP clade [65], which had diverged over a similar timescale as the neotropical bamboos. On the basis of this characteristic, neotropical bamboos represent a distinct lineage that differs from the paleotropical bamboos; however, the evolutionary causes of these mutation events are not very clear.

### Divergence time of Bambuseae

Considerable variation in the dates of bamboo history exists among published works [42, 66]. Divergence date estimates for nodes using molecular sequences rely heavily on phylogeny, dating algorithms and calibration strategies. Previous studies of bamboos were heavily based on one or several DNA segments [42, 67], which may be insufficient to estimate the true branch lengths. To compensate for this deficiency, the present study applied complete chloroplast genome sequences to reconstruct the evolutionary history of bamboos. Compared with earlier genome-scale calibrated analyses of 12 bamboos with 3 calibration points [68], our molecular dating results are slightly younger. This result might also reflect the impact of the use of different calibration dates. We employed a new Pliocene macrofossil as a constraint for the *Guadua* crown group instead of using only secondary calibration points. Most bamboo genera appear to have diverged after the Miocene, and speciation events of extant species appear to have occurred during or after the Pliocene. Some nodes had large credibility intervals, possibly due to the use of fewer calibrations and incomplete sampling. Given that our estimated time accords with the bamboo fossil evidence at the time of the Miocene [69, 70], the resulting molecular divergence time of bamboos might represent a reasonable estimate despite the large divergence time intervals of some nodes.

## Supporting Information

S1 Table *G*. *angustifolia*-specific primers. (XLSX)

S2 Table Taxa included in the analyses, with GenBank numbers and references. (XLSX)

S3 Table Tajima’s relative tests for 76 coding genes in the three compared bamboos. Genes that evolve at similar rates are shown in bold. P<0.05 indicates rates that are significantly different. GA: *Guadua angustifolia*; CS: *Cryptochloa strictiflora*; DL: *Dendrocalamus latiflorus*.
(XLSX)

S4 Table dN, dS and dN/dS values of individual genes. dN: nonsynonymous substitution rates; dS: synonymous substitution rates; dN/dS: ratio of nonsynonymous to synonymous substitutions.
(XLSX)

S5 Table Divergence times for the most recent common ancestor of the clades recovered in this study. (XLSX)
